# Use of Low-Cost Sensors to Study Atmospheric Particulate Matter Concentrations: Limitations and Benefits Discussed through the Analysis of Three Case Studies in Palermo, Sicily

**DOI:** 10.3390/s24206621

**Published:** 2024-10-14

**Authors:** Filippo Brugnone, Luciana Randazzo, Sergio Calabrese

**Affiliations:** 1 Dipartimento di Scienze della Terra e del Mare, Università degli Studi di Palermo, Via Archirafi, 36, 90123 Palermo, Italy; filippo.brugnone@unipa.it; 2 Istituto Nazionale di Geofisica e Vulcanologia, Sezione di Palermo, Via Ugo la Malfa, 153, 90146 Palermo, Italy

**Keywords:** particulate matter, air pollution, low-cost sensors, Saharan dust, wood smoke, fireworks

## Abstract

The paper discusses the results of the concentrations of atmospheric particulate matter, in the PM_2.5_ and PM_10_ fractions, acquired by two low-cost sensors. The research was carried out from 1 July 2023 to 30 June 2024, in Palermo, Sicily. The results obtained from two systems equipped with the same sensor model were compared. Excellent linear correlation was observed between the results, with differences in measurements falling within instrumental accuracy. Two instruments equipped with different sensors, models Novasense SDS011 and Plantower PMSA003, were placed at the same site. These were complemented by a weather station to measure meteorological parameters. Upon comparing the atmospheric particulate matter concentrations measured by the two instruments, it was observed that there was a good linear correlation for PM_2.5_ and a poor linear correlation for PM_10_. Additionally, the PMSA003 sensor appeared to consistently record higher concentrations than the SDS011 sensor. During periods influenced by natural sources and/or anthropogenic activities at the regional and/or local scale, i.e., the dispersal of Saharan sands, forest fires, and local events using fireworks, abnormal concentrations of atmospheric particulate matter were detected. Despite the inherent limitations in precision and accuracy, both low-cost instruments were able to identify periods with abnormal concentrations of atmospheric particulate matter, regardless of their source or type.

## 1. Introduction

Air pollution is a significant threat to public health as our society continues to evolve and the sources of pollution in the environment continue to grow. This is especially prominent in densely populated areas with public transportation, factories, and other urban facilities. The World Health Organization [1] estimates that approximately 7 million people die from the effects of air pollution every year. Air pollution is responsible for causing heart disease, strokes, lung cancer, and chronic respiratory diseases [2,3,4]. Nine out of ten people breathe air that exceeds the WHO’s recommendations for air pollutants, impacting individual health and placing a burden on the healthcare system and the broader economy [5]. Urban areas are the focus of contemporary environmental research due to the concentration of atmospheric particulate matter (PM). Understanding PM concentration dynamics is important for devising effective mitigation strategies and informing policy decisions and public health initiatives.

The assessment of air quality is mainly performed using expensive monitoring stations, which are only deployed in a few locations per city due to their high cost and maintenance expenses. These monitoring stations are typically found in densely populated areas or near city centers, resulting in large geographic areas with no coverage. This leads to limited and less accurate air quality information, especially in areas far from monitoring stations where the data are generalized over a wider area. To address this limited coverage, there has been a shift toward using wireless sensors in public vehicles like buses, trams, and trains to monitor air quality [6]. However, the sensory readings are confined to the routes of these vehicles [7,8]. To improve the monitoring strategies, efforts have been made to leverage the power of the public by using portable and low-cost air quality sensors to collect high-resolution air quality data. In the last ten years, many scientific researchers have emphasized the limitations and potential of low-cost sensors for monitoring air quality. Over time, low-cost air pollution sensors have shown an improved ability to gather accurate, real-time measurements with high spatial and temporal resolution, leading to their development on a global scale [9,10]. Particulate matter measurements from several air quality monitoring networks worldwide have been made publicly available in recent years [11].

Particulate matters (PMs) are categorized based on the size of the particles into PM_2.5_ and PM_10_, representing their maximum diameter in micrometers. Exposure to particulate matter (PM), especially particles less than 10 μm in diameter, poses significant health risks. These small particles can penetrate deep into the lungs and even enter the bloodstream, leading to various health problems. Scientific studies have linked PM exposure to several adverse effects, including (i) premature death in individuals with heart or lung disease [12]; (ii) nonfatal heart attacks and irregular heartbeat [12]; (iii) aggravated asthma and decreased lung function [12]; and (iv) increased respiratory symptoms, such as airway irritation, coughing, or difficulty breathing [12]. Particularly vulnerable groups include people with pre-existing heart or lung diseases, children, and older adults. The environmental impact of PM also extends to visibility impairment, ecosystem diversity alteration, and material damage, which can have broader implications for public health and welfare [12]. Understanding the health effects associated with PM exposure is crucial for developing policies and strategies to protect public health against PM pollution. Thankfully, the inhaled dosage of these pollutants and their health effects can be estimated due to their physical characteristics. For example, studies have shown that heart rate variability is connected to the inhaled PM dose in the lungs [13]. Additionally, PM_2.5_, which is inhaled both outdoors and indoors, has been associated with high blood pressure, and computing the inhaled PM dosage also aids in assessing cardiovascular effects [14].

This study provides a comprehensive assessment of PM levels and sources in Palermo City (Sicily, Italy), using commercial use and low-cost sensors, for one year, starting in July 2023. Palermo is densely populated, with an industrial and a port area of moderate extension, in a context where the contributions of particulate matter from natural and anthropogenic sources overlap. The main natural sources of particulate matter in the atmosphere are soil erosion and dust transport from other regions of the Mediterranean basin (e.g., Saharan sand), and aerosols of marine origin and ash released during fires are other important natural sources. In addition, the main anthropogenic sources of particulate matter in the atmosphere are activities typical of a densely populated area, such as road and sea traffic, domestic heating, and wood burning. The article will illustrate the comparison between the two different sensors used. Furthermore, three case studies will be subjected to a detailed examination: the effect of Saharan sand transport within the Mediterranean basin, associated with strong winds from the southern quadrants; the impact of extensive forest fires on the air quality of the surrounding area; and the effects on air quality of fireworks associated with New Year’s Eve celebrations. The findings are expected to serve as a benchmark to assess the impact on air quality of different natural and/or anthropogenic phenomena and as a guide for recognizing them.

## 2. Materials and Methods

### 2.1. Study Location

**Figure 1:**
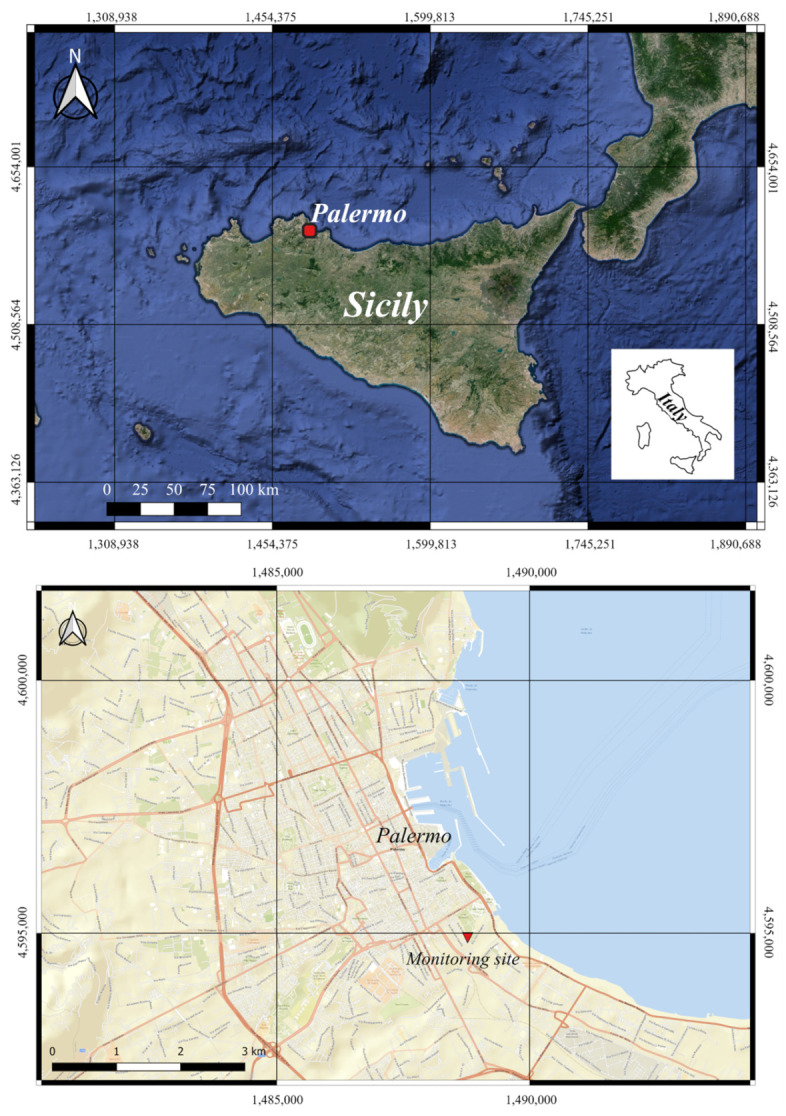
Location of the study area, Base map: Google Earth. Coordinate system: WGS84 EPSG 3857. Made with Quantum Gis v. 3.36.3 “Maidenhead”, distributed under the GNU General Public License.

The study was carried out for one year, from 1 July 2023 to 30 June 2024, in the metropolitan area of Palermo, a city of 628,348 inhabitants [15], located on the northwest coast of Sicily, in the middle of the Mediterranean Basin (Figure 1). The study area belongs to a geologically complex area. The metropolitan area of Palermo is surrounded by mountains, which result from the piling up of deep-water and carbonate platform tectonic units (Imerese and Panormide) [16]. The Palermo urban area is densely populated and serves as a significant cultural and commercial hub in Sicily. The primary contributors to gas and atmospheric particulate matter emissions are vehicle traffic, domestic heating, and ships, especially cruise ships that frequently crowd the city’s main port, particularly in the summer months. The measurements were carried out from the roof (about 20 m from the ground and 33 m above sea level) of the “Emilio Segré” building in “Via Archirafi no. 36” (Dipartimento di Scienze della Terra e del Mare, Università degli Studi di Palermo). The locations of the sampling site where the systems for monitoring the PM concentration were deployed are shown in Figure 1 and presented in Table S1.

### 2.2. Data Collection

**Figure 2:**
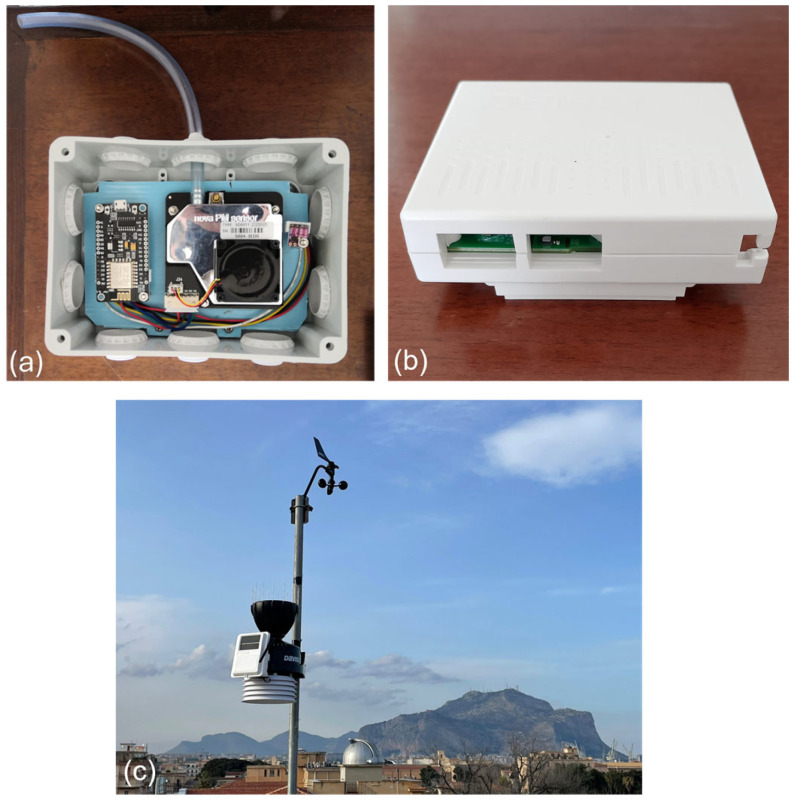
Inovafit SDS011 (**a**) and Davis Instruments Corporation “AirLink” (**b**) air quality monitoring systems, and the Davis Instruments Corporation “Vantage Pro2” weather station (**c**) installed on the roof of the “Emilio Segré” building in “Via Archirafi no. 36” (Dipartimento di Scienze della Terra e del Mare, Università degli Studi di Palermo).

The measurement of atmospheric particulate matter concentrations in the PM_2.5_ and PM_10_ classes was carried out using two different sensors (Figure 2a). Particulate matter sensors were selected from a range of commercially available solutions primarily based on promising results of the validation tests carried out in the laboratory and real conditions [17,18,19,20].

The first one was a very low-cost sensor, the SDS011 [21]. It is a quite recent air quality sensor developed by Inovafit, a spin-off from the University of Jinan in the Shandong province, China [22]. The technology is based on laser diffraction theory, where particle density distribution is specified from the light intensity distribution patterns [20,23]. This PM sensor comes compact, is light, has low energy consumption, operates at a high sampling frequency, and costs about one hundred euros. The sensor contains a digital output and a built-in fan, which can detect particles with a minimum diameter of 0.3 μm and measure concentrations from 0.0 to 999.9 μg m^−3^. A built-in algorithm converts the particle density distribution into particle mass in μg m^−3^ (PM_2.5_ and PM_10_). In terms of accuracy, several studies have been conducted. A study by Božilov et al., 2022 [24], showed that the SDS011 sensor demonstrated high linearity in comparison with PM_10_ and PM_2.5_ concentrations measured in outdoor air with reference-equivalent instrumentation, with R^2^ values ranging from 0.52 up to 0.83. In addition, very good agreement (R^2^ values ranging from 0.93 up to 0.97) with the gravimetric PM_10_ and PM_2.5_ method was obtained in the indoor environment (30% < relative humidity < 70%). However, high relative humidity (over 70%) negatively affected the PM monitors’ response, especially in the case of PM_10_ concentrations (high overestimation). Indeed, condensation of water vapor falsifies readings. In our study, we also compared the results of two SDS011 sensors to see if they measured comparable concentrations of PM_2.5_ and PM_10_. A short technical specification of the SDS011 is given in Table S2.

The second one was a Davis Instruments Corporation (Diablo Avenue, Hayward, California) “AirLink” air quality monitoring system. The “AirLink” sensor is an air quality monitoring device that provides real-time measurements of PM_1.0_, PM_2.5,_ and PM_10_ mass concentrations, global air quality indices (AQI), and environmental parameters such as temperature, dew point, wet bulb temperature, heat index, and relative humidity. Data can be visualized and accessed on the dashboard and mobile applications. The “AirLink” sensors can be used as stand-alone sensors or paired with a Davis weather station. The Davis Instruments Corporation “AirLink” sensors use a PMSA003 laser particle counter, where laser beams detect particles by their reflectivity. These sensors count suspended particles in sizes of 0.3, 1.0, 2.5, and 10 μm. These particle counts are processed by the sensor using a complex algorithm to calculate the PM_1.0_, PM_2.5,_ and PM_10_ mass in μg m^−3^. The PMSA003 sensors come factory-calibrated. The Davis Instruments Corporation “AirLink” sensor measures particulates with an accuracy of ±10 μg m^−3^ in concentrations [25]. In a laboratory evaluation conducted by the South Coast Air Quality Management District, the “AirLink” sensors showed high accuracy (92.3% to 97.8%) for all tested PM_2.5_ concentrations compared to the reference FEM T640x for the entirety of the test [26]. The sensors tended to overestimate PM_2.5_ concentration values at lower levels while underestimating at higher levels compared to the FEM T640x PM_2.5_ mass concentration at 20 °C and 40% relative humidity [26]. The “AirLink” sensors also showed very strong correlations with the FEM T640x mass concentration PM_2.5_ (R^2^ > 0.99) [26]. Overall, the “AirLink” sensors demonstrated high precision for all combinations of PM_2.5_ concentration, temperature, and relative humidity [26]. However, low-to-moderate PM_2.5_ concentration variations were observed among the three “AirLink” sensors at 20 °C and 40% relative humidity [26]. These results are based on specific tests and actual performance may vary depending on environmental conditions and other factors. The basic characteristics of the sensor are presented in Table S2.

Both measurement systems with PM sensors make it possible to record PM concentration changes in short time intervals. The basic set used for analysis consists of averaged PM_2.5_ concentrations over 1 min intervals.

Both systems were equipped with high-performing temperature and relative humidity sensors. The SDS011 was fitted with a Sensirion SHT30-DIS-B temperature and humidity sensor, which has a typical relative humidity accuracy of ±2% over the range of 10–90%, and a typical temperature accuracy of ±0.2 °C in the range of 0–65 °C [27]. On the other hand, the Davis Instruments Corporation “AirLink” used a Sensirion SHT31-DIS-B for temperature and relative humidity measurements, with a typical relative humidity accuracy of ±2% over the entire measuring range (0–100%), and typical temperature accuracy of ±0.2 °C in the range of 0–90 °C [28]. Additionally, other meteorological parameters such as wind speed and direction at a height of 2 m, as well as rainfall, were measured using a Davis Instruments Corporation “Vantage Pro2” weather station (Figure 2b). This professional weather station offers a wide range of features and robust performance. The temperature and relative humidity of the atmosphere were also measured using a sensor identical to that installed in the Davis Instruments Corporation “AirLink”.

## 3. Results and Discussion

### 3.1. Comparison of Temperature and Relative Humidity Measurements

**Figure 3:**
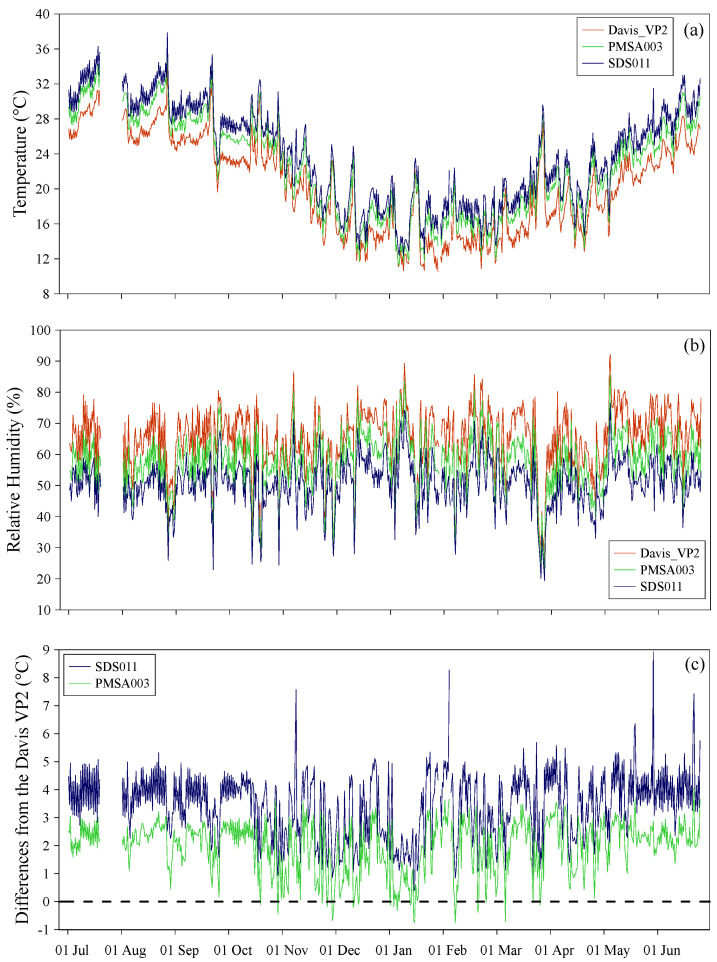
(**a**) Values of atmospheric temperature (°C) and (**b**) relative humidity (%) measured by the three different instruments from 01 July 2023 to 30 June 2024: Davis Instruments Corporation “Vantage Pro2” (red), PMSA003 (green), SDS011 (blue). (**c**) Temperature differences (°C) between SDS011 and Davis Instruments Corporation “Vantage Pro2” (blue), and between PMSA003 and Davis Instruments Corporation “Vantage Pro2” (green). The dotted line represents the reference of the measured temperature values (°C) of the Davis Instruments Corporation “Vantage Pro2” weather station.

The temperature (°C) and relative humidity (%) measurements of the atmosphere were taken during the study period using air quality monitoring devices (SDS011 and Davis Instruments Corporation “AirLink”) and the Davis Instruments Corporation “Vantage Pro2” professional weather station. The average values, on a 12 h basis, measured by the three measurement systems, are shown in Figure 3a,b.

Below are the ranges and average temperatures (in brackets) measured by the Davis Instruments Corporation “Vantage Pro2”, PMSA003, and SDS011, respectively: 10.4–35.4 °C (20.3 °C), 11.3–36.7 °C (22.2 °C), and 12.4–37.9 °C (23.8 °C). For the relative humidity (%) the values were 22–92% (65%), 22–86% (58%), and 19–76% (51%).

When comparing the temperature and relative humidity trends from the three different systems, there was a strong overall agreement. However, we noticed significant temperature differences, both in extreme values and averages. These differences can be attributed to two main reasons: (i) the SDS011 and PMSA003 systems were installed on a wall with an eastern/northeastern exposure. The heating of the wall surface during the day, especially on the sunniest days, affected the temperature readings of these two instruments; (ii) both air quality monitoring systems did not have sufficient shielding against solar radiation. The radiant heat problem is clearly visible when looking at the temperature differences between the SDS011 instrument (University of Jinan, Shandong province, China) and the Davis Instruments Corporation “Vantage Pro2” weather station (Davis Instruments Corporation, Diablo Avenue, Hayward, California), and between the same weather station and the PMSA003 instrument (Figure 3c). For almost the entire study period, the temperature values measured by the two low-cost sensors were higher than those measured by the reference weather station. The location and hardware characteristics of these systems were not suitable for accurate temperature measurements, leading to an overestimation of the readings. According to the standards set by the World Meteorological Organization for measuring atmospheric temperature, it is important to position the instrument away from heat sources and surfaces that could affect measurement accuracy [29]. Additionally, it is crucial to shield the instrument from direct solar radiation and reflections. The Davis Instruments Corporation “Vantage Pro2” weather station was installed in compliance with these standards. In addition, as declared by the producer, this weather station is equipped with a solar radiation shield that received a five-star rating from the World Meteorological Organization [30]. Therefore, the temperature values measured by this instrument were the most accurate and will be considered in the following discussion. Similar considerations apply to the relative humidity values measured for the atmospheric temperature measurements. As this parameter is inversely related to temperature, the overestimation of temperature attributed to the SDS011 and PMSA003 instruments led to an underestimation of the relative humidity values measured by the same instruments. The most accurate values were found in the recordings by the Davis Instruments Corporation “Vantage Pro2” weather station, and these will be considered in further discussion. The test thus proved that incorrect positioning of the instruments and the absence, in the SDS011 and PMSA003 measuring systems, of adequate shielding against solar radiation resulted in inaccurate measurements of atmospheric temperatures and related parameters, i.e., relative humidity.

### 3.2. Comparison between Two Different SDS011 Sensors

**Figure 4:**
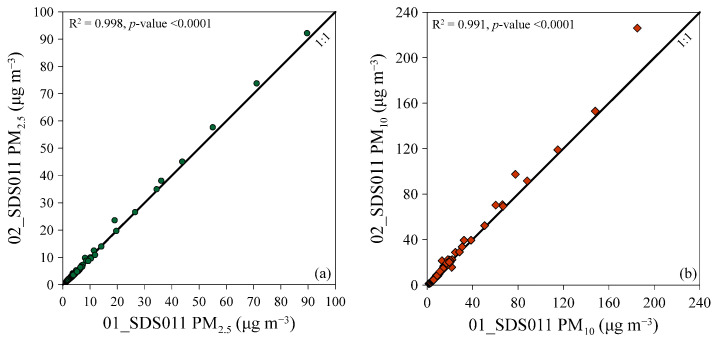
Correlation of PM_2.5_ (**a**) and PM_10_ (**b**) concentration measurements between two different SDS011 sensors (01_SDS011 and 02_SDS011). The solid lines are the 1:1 ratio between the two different sensor readings.

Before the start of the research, we conducted a 28-day comparison between two different SDS011 sensors, to ensure consistent sensor response. This comparison occurred outdoors and encompassed measurements for both PM_2.5_ and PM_10_ concentrations. Concentration values were simultaneously measured by both instruments at 1 min intervals. The comparison between the two sensors was based on the two-hour averages of the measured values, in which 294 values were compared for both particle size classes. An excellent linear correlation was observed between the two sensors, with a coefficient of determination R^2^ of 0.998 (*p*-value < 0.0001) for PM_2.5_ (Figure 4a) and 0.991 (*p*-value < 0.0001) for PM_10_ (Figure 4b).

### 3.3. Comparison between PMSA003 and SDS011 Sensors

**Figure 5:**
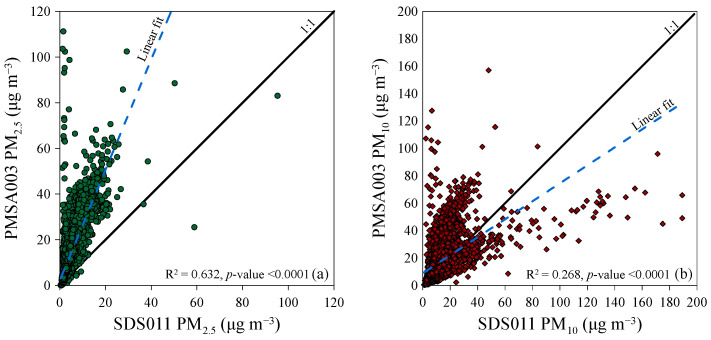
Correlation of PM_2.5_ (**a**) and PM_10_ (**b**) concentration measurements by SDS011 and PMSA003 sensors considering data from the entire sampling period (July 2023–June 2024). The solid thick lines are the 1:1 ratio between the two sensor readings. The blue dotted line represents the linear correlation line between the measurements of the two sensors.

After confirming the consistent readings from the two different sensors, the system equipped with the “01_SDS011” sensor was installed at the monitoring site. This was supported by the Davis Instruments Corporation “AirLink” air quality monitoring system. Both systems measured concentrations of airborne particulate matter, with a frequency of one measurement per minute from 1 July 2023 to 30 June 2024. The comparison between the two measurement systems over the entire monitoring period was made by comparing the arithmetic averages of the 8783 values measured in each 1 h interval. For the fraction PM_2.5_, it was observed that the values measured by the Davis Instruments Corporation “AirLink” air quality monitoring system (i.e., the PMSA003 sensor) were almost always higher than those measured by the SDS011 sensor (~95% of the cases). The PM_2.5_ median concentrations were 3.5 μg m^−3^ and 10 μg m^−3^ for the SDS011 and the PMSA003, respectively, with a ratio of 2.99, and an average difference of +8.9 μg m^−3^ between the two sensors. The correlation between the PM_2.5_ concentration measurements of the two instruments was relatively strong, with a coefficient of determination R^2^ of 0.632 (*p*-value < 0.0001) (Figure 5a). When considering the PM_10_ fraction, it was observed that in ~63% of the cases, the reading from the sensor PMSA003 was higher than that of the SDS011 sensor. The PM_10_ median concentrations were 9.6 μg m^−3^ and 13 μg m^−3^ for the SDS011 and the PMSA003, respectively, with a ratio of 1.3, and an average difference of +4.9 μg m^−3^ between the two sensors. There was almost no linear correlation between the readings of the two instruments (R^2^ = 0.268, *p*-value < 0.0001) (Figure 5b). Therefore, the best correlations were observed in the measurements of PM_2.5_ concentration compared to PM_10_. Effectively, all low-cost sensors for particulate matter monitoring only measure up to 1.5 or 3 µm, they do not measure up to 10 µm. They display PM_10_, which is usually calculated from PM_2.5_ and based on Arizona Road Dust. The use of a different calculation algorithm between the two sensors could be the reason for the poor correlation between the values returned for this parameter, unlike what was observed for the PM_2.5_ fraction, which is measured directly.

Briefly, the PMSA003 sensor often returned higher values than the SDS011 for both particle size fractions. Although both sensors are marketed as “factory calibrated”, the results observed during the research would suggest a different calibration of the two sensors or a lack of calibration of one or both sensors. Before conducting field measurements, it would be essential to verify the calibration of these low-cost sensors by making measurements in controlled atmosphere chambers (with known concentrations of particulate matter in the air) or by comparison with professional instruments whose calibration is known [31]. Additionally, the poor linear correlation between the measurements made by the two instruments throughout the monitoring period meant that one or both sensors used could not measure the true concentration of the two fractions of particulate matter.

### 3.4. The Saharan Sand and Fire Ash Study Case

**Figure 6:**
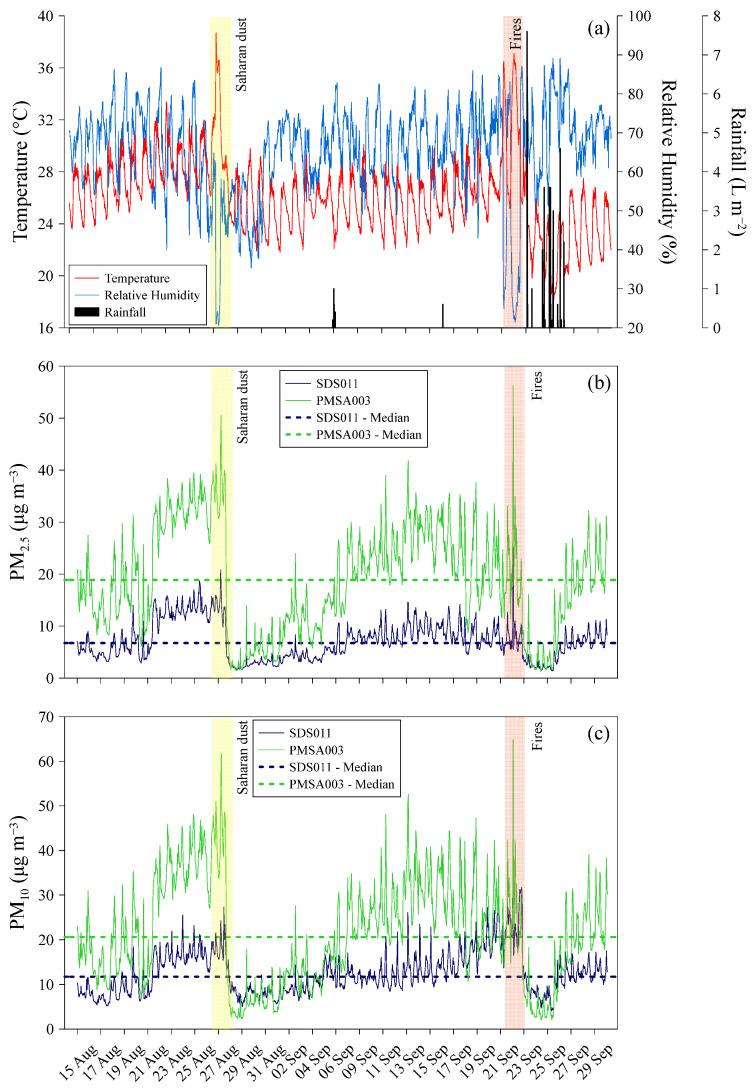
Temperature (°C), Relative Humidity (%), and Rainfall (L m^−2^) (**a**) measured by the Davis Instruments Corporation “Vantage Pro2”, PM_2.5_ (**b**) and PM_10_ (**c**) hourly arithmetic average concentrations (μg m^−3^) measured by the SDS011 (blue lines) and by the PMSA003 (green lines), between 15 August 2023 and 30 September 2023 in Palermo. The yellow-shaded area indicates the dispersion period of Saharan sand at the end of August 2023. The red-shaded area indicates the dispersion period of Saharan sand associated with the dispersion of ash from fires at the end of September 2023.

Between 26 and 28 August 2023, a North African anticyclone moved into the Mediterranean basin, causing winds to shift clockwise in the region. As a result, southerly winds carried large amounts of Saharan sand across the Mediterranean. Locally, the activation of adiabatic winds, known as “Foehn”, led to a significant increase in temperature and a sharp decrease in relative humidity in Palermo, with values reaching up to 38.7 °C and 22%, respectively (Figure 6a).

**Figure 7:**
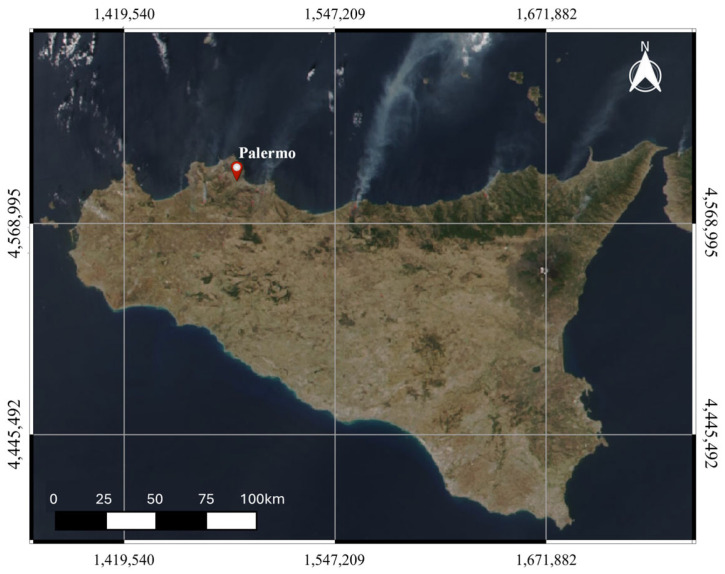
Satellite image of Sicily taken by the satellite Moderate Resolution Imaging Spectroradiometer (MODIS)—Visible Infrared Imaging Radiometer Suite (VIIRS)—NASA S-NPP and NOAA20, on 22 September 2023. In opaque white, clouds of water vapor are visible. In semi-transparent white, dispersed in a south-east/north-west direction, are visible the ash clouds generated by the forest fires that affected various areas of northern Sicily on 22 September 2023.

The dispersal of Saharan sand into the atmosphere resulted in a decline in air quality, as indicated by the high levels of both PM_2.5_ and PM_10_ measured by the PMSA003 and the SDS011 sensors. Between 26 and 28 August 2023, hourly arithmetic average concentrations of up to 21 μg m^−3^ and 27 μg m^−3^ for PM_2.5_ and PM_10_, respectively, were measured by the sensor SDS011 (Figure 6b,c). Higher arithmetic average concentrations, reaching up to 51 μg m^−3^ and 62 μg m^−3^, were measured by the sensor PMSA003 during the same time frame and for the same particle sizes (Figure 6b,c). Although different, both sensors returned median values during this period that were significantly above their median values from the one year of monitoring. In the days following this event, the settling of sand led to atmospheric particulate matter concentrations returning to near-normal levels. The subsequent weeks were characterized by stable weather conditions, including light and mostly breezy winds, with only one rain event on the night of 5–6 September, resulting in a gradual increase in atmospheric particulate matter concentrations. A new anticyclonic phase with characteristics similar to those at the end of August affected the Mediterranean from the evening of 20 September to the morning of 23 September. In Palermo, temperatures rose to 37.1 °C and relative humidity dropped to 22% (Figure 6a). Once again, the impact of Saharan sand on air quality was observed by both particulate matter sensors, likely combined with the dispersion of ash from significant forest fires near Palermo (Figure 7). As reported in the local and national news [32], the city of Palermo was hit by several fires, particularly on 22 September. These fires, together with those that occurred in July of the same year, burned 4314 hectares in the municipality of Palermo alone, almost 3% of the municipality’s territory, according to EFFIS, the European Forest Fire Information System [33]. The consequences and damage caused by the fires were numerous. Dozens of cars and several industrial sheds were burnt. The flames also reached the *Bellolampo* rubbish dump. Other fires forced the evacuation of some buildings on the university campus in *Viale delle Scienze* and the evacuation of about twenty families from other buildings threatened by the flames. Several stretches of road were closed to traffic for several hours and some schools were also closed.

Between the evening of 20 September to the morning of 23 September, hourly arithmetic average concentrations of up to 25 μg m^−3^ and 41 μg m^−3^ for PM_2.5_ and PM_10_, respectively, were measured by the sensor SDS011 (Figure 6b,c). Higher arithmetic average concentrations, reaching up to 56 μg m^−3^ and 65 μg m^−3^, were measured by the sensor PMSA003 during the same time frame and for the same particle sizes (Figure 6b,c). During this heat wave, the hourly median values measured by both sensors were comparable to or slightly higher than those at the end of August. They were also well above the median values observed during the yearlong monitoring. The heat wave ended abruptly with a rain event on the afternoon of 23 September 2023. The meteorological station used in our research measured an accumulation of 22.8 L m^−2^ of rain in just 60 min. During the most intense phase of the rainfall, the rain intensity reached a value of 123.8 L m^−2^ per hour. This event led to a sudden drop in temperature of up to 22.5 °C and an increase in relative humidity of up to 91% (Figure 6a). Additionally, the rain caused a sudden deposition of atmospheric particulate matter in suspension, through the scavenging process, which was detected by both particulate sensors. Following the rain, the PM_2.5_ concentration dropped to 2.5 μg m^−3^ and 3.9 μg m^−3^ for SDS011 and PMSA003, respectively, while the PM_10_ hourly arithmetic average concentrations decreased to 8.4 μg m^−3^ and 5.1 μg m^−3^ for the same sensors (Figure 6b,c). It is important to remember that, as stated in the manufacturer’s datasheet, the correct functioning of the SDS011 sensor is guaranteed up to 70% relative humidity.

**Figure 8:**
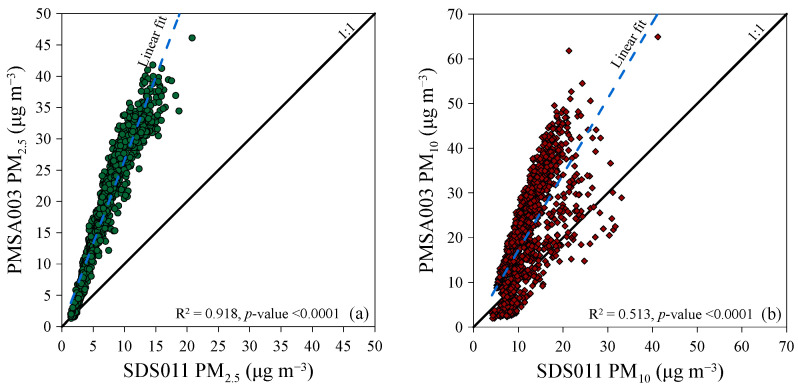
Correlation of PM_2.5_ (**a**) and PM_10_ (**b**) hourly median concentration measurements by SDS011 and PMSA003 sensors from 15 August 2023 to 30 September 2023 in Palermo. The solid thick lines are the 1:1 ratio between the two sensor readings. The blue dotted line represents the linear correlation line between the measurements of the two sensors.

Notwithstanding, while there was a low correlation between the atmospheric particulate matter concentrations measured by the two sensors over the entire monitoring period, there was a stronger correlation during the specific period from 15 August 2023 to 30 September 2023. For PM_2.5_ measurements, a coefficient of determination (R^2^) of 0.918 (*p*-value < 0.0001) was calculated (Figure 8a), and for PM_10_ hourly median concentration (Figure 8b), the coefficient of determination R^2^ between the two instruments was 0.513 (*p*-value < 0.0001). Therefore, the best correlations were observed in the measurements of PM_2.5_ concentration compared to PM_10_. When considering the period covered by the case studies, the PMSA003 sensor measured higher median values than the SDS011 sensor in ~98% and ~80% of the cases for PM_2.5_ and PM_10_, respectively.

### 3.5. The Case Study of New Year’s Eve Fireworks

The last case study presented in this paper examines the impact of New Year’s Eve fireworks on air particulate matter concentration in Palermo.

**Figure 9:**
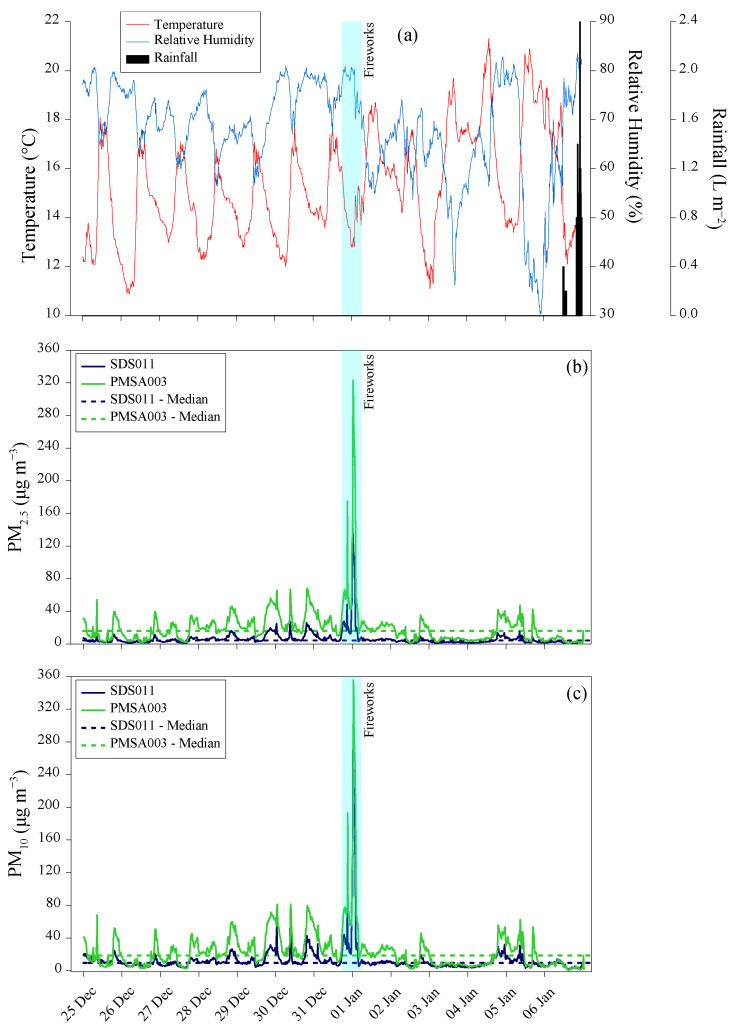
Hourly Temperature (°C), Relative Humidity (%), and Rainfall (L m^−2^) values measured by the Davis Instruments Corporation “Vantage Pro2” (**a**), and PM_2.5_ (**b**) and PM_10_ (**c**) 15 min arithmetic average concentrations (μg m^−3^) measured by the SDS011 (blue lines) and by the PMSA003 (green lines), respectively, between 25 December 2023 and 6 January 2024 in Palermo. The cyan-shaded area indicates the firework shows period.

From 25 December 2023 to 6 January 2024, the weather was stable due to the presence of the Azores anticyclone over the Mediterranean. This resulted in temperatures ranging from 11 °C to 21 °C, with light winds and no rain (Figure 9a).

Regarding particulate matter concentrations in the atmosphere, measurements from the SDS011 sensor showed 15 min arithmetic average values between 0.6 μg m^−3^ and 27 μg m^−3^ (median 3.6 μg m^−3^) for PM_2.5_ and between 1.3 μg m^−3^ and 58 μg m^−3^ (median 9.1 μg m^−3^) for PM_10_ for the periods between 25 and 30 December 2023 and between 2 and 6 January 2024 (Figure 9b,c).

As highlighted for the other case studies, higher median concentrations were measured by the PMSA003 sensor, ranging from 0.2 μg m^−3^ to 68 μg m^−3^ (median 13 μg m^−3^) for PM_2.5_ and from 0.6 μg m^−3^ to 81 μg m^−3^ (median 15 μg m^−3^) for PM_10_ in the same periods (Figure 9b,c).

It is worth noting that New Year’s Eve celebrations, particularly the midnight fireworks on 31 December 2023, contribute to increased particulate matter in the atmosphere. Between 31 December 2023, and 1 January 2024, the sensors measured 15 min arithmetic average concentrations of PM_2.5_ up to 151 μg m^−3^ and 323 μg m^−3^ by SDS011 and PMSA003, respectively. For PM_10_, the sensors measured 15 min arithmetic average concentrations up to 270 μg m^−3^ and 356 μg m^−3^ during the same period. Both peaks occurred between 17 UTC on 31 December and 02 UTC on 1 January, coinciding with the peak of the fireworks. Subsequently, the concentrations of both fractions returned to their pre-firework values (Figure 9b,c).

**Figure 10:**
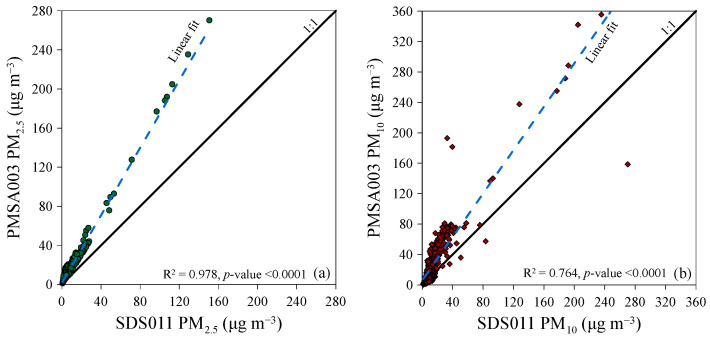
Correlation of PM_2.5_ (**a**) and PM_10_ (**b**) 12 h median concentration measurements by SDS011 and PMSA003 sensors from 25 December 2023 to 6 January 2024 in Palermo. The solid thick lines are the 1:1 ratio between the two sensor readings. The thin solid lines define the instrumental accuracy range (±10 μg m^−3^). The blue dotted line represents the linear correlation line between the measurements of the two sensors.

Consistently across the entire research period, from 25 December 2023 to 6 January 2024, the ratio of medians between the concentrations measured by the two sensors was 3.6 for PM_2.5_ and 1.9 for PM_10_ (Figure 10). During this short period of observation, both sensors showed a strong correlation in their measurements of particulate matter concentration, with coefficients of determination (R^2^) of 0.978 (*p*-value < 0.0001) for PM_2.5_ and 0.764 (*p*-value < 0.0001) for PM_10_. Therefore, the best correlations were observed in the measurements of PM_2.5_ concentration compared to PM_10_.

In summary, despite the differences in measured concentrations due to calibration issues and to the different built-in algorithms used to convert the particle density distribution into particle mass in μg m^−3^, both sensors were effective in identifying abnormal levels of PM_2.5_ and PM_10_ associated with fireworks and other natural or human activities. This suggests that these low-cost sensors can detect unusual particulate matter concentrations, regardless of their origin or composition.

## 4. Conclusions

Between 1 July 2023 and 30 June 2024, pilot research was conducted in Palermo to compare two low-cost instruments used for measuring atmospheric particulate matter concentration. These instruments were equipped with different sensor models: SDS011 and PMSA003. The research found that incorrect positioning of the instruments and the absence, in the SDS011 and PMSA003 measuring systems, of adequate shielding against solar radiation resulted in inaccurate measurements of atmospheric temperatures and related parameters, i.e., relative humidity. Before the monitoring campaign began, the results from two identical instruments developed by Inovafit, a spin-off from the University of Jinan in the Shandong province, China, and equipped with the same sensor (SDS011), were compared. The comparison showed a strong linear correlation between the concentrations measured by the two instruments. The determination coefficients (R^2^) were 0.998 (*p*-value < 0.0001) for PM_2.5_ and 0.991 (*p*-value < 0.0001) for PM_10_. Later, one of these instruments was used alongside a Davis Instruments Corporation “Airlink”, equipped with a different sensor, model PMSA003. The comparison of the atmospheric particulate matter concentrations measured by the two instruments showed a good linear correlation for PM_2.5_, with determination coefficients (R^2^) equal to 0.632 (*p*-value < 0.0001), and a poor correlation for the calculated fraction PM_10_, with a R^2^ equal to 0.268 (*p*-value < 0.0001). Additionally, in ~95% of cases for PM_2.5_ and ~63% of cases for PM_10_, the values measured by the PMSA003 sensor were higher than those measured by the SDS011 sensor. Possible reasons for the observed differences could be the failure or incorrect calibration of one or both sensors and the different built-in algorithms used to convert the particle density distribution into particle mass in μg m^−3^. During periods influenced by natural and/or human activities at the regional and/or local scale, i.e., the dispersal of Saharan sands, forest fires, and parties involving fireworks, abnormal median concentrations of atmospheric particulate matter were detected, up to an order of magnitude greater than the median values measured in the other periods. Hourly arithmetic average concentrations up to 21 μg m^−3^ (SDS011) and 51 μg m^−3^ (PMSA003) for PM_2.5_, and up to 27 μg m^−3^ (SDS011) and 62 μg m^−3^ (PMSA003) for PM_10_ were calculated in the August–September 2023 case studies examined in the paper. Fifteen-minute arithmetic average concentrations up to 151 μg m^−3^ (SDS011) and 323 μg m^−3^ (PMSA003) for PM_2.5_ and up to 270 μg m^−3^ (SDS011) and 356 μg m^−3^ (PMSA003) for PM_10_ were measured in the case study of New Year’s Eve fireworks. In all three case studies, the atmospheric particulate matter concentrations measured by the two sensors showed better linear correlations compared to the entire dataset. The best correlations were observed in the measurements of PM_2.5_ concentration compared to the calculated PM_10_ for the whole study period.

Despite the inherent limitations in precision and accuracy, especially in the absence of proper calibration, both low-cost instruments were able to identify periods with abnormal concentrations of atmospheric particulate matter, regardless of their source or type.

## Data Availability

Research data are already included in the manuscript.

## Supplementary Materials

The following supporting information can be downloaded at: https://www.mdpi.com/article/10.3390/s24206621/s1, Table S1: Study site location; Table S2: Basic characteristics of the SDS011 and the PMSA003 sensors.
