# Supplementary material for: Use of Low-Cost Sensors to Study Atmospheric Particulate Matter Concentrations: Limitations and Benefits Discussed through the Analysis of Three Case Studies in Palermo, Sicily
Source: Sensors (Basel). 2024 Oct 14;24(20):6621. doi: 10.3390/s24206621 (PMC11511236; doi:10.3390/s24206621)
Supplement: Supplementary file 1 [file sensors-24-06621-s001.zip › sensors-3238643-supplementary.pdf]

Supplementary Materials

Table S1. – Study site location

Site	Location	Type of site	Position WGS84 EPSG 3857	Altitude (m a.s.l.)	Elevation from the roof (m)	Elevation from the ground (m)
Palermo	Via Archirafi, 36	Urban	1488650.8 4594937.5	33	2	22

Table S2. – Basic characteristics of the SDS011 and the PMSA003 sensors.

Parameter	Value	Value
Model	SDS011	PMSA003
Detection range	0.0 a 999.9 μg m^{-3}	0.0 a 999.9 μg m^{-3}
Response time	≤ 10 s	≤ 10 s
Uncertainties	Maximum of ± 15% and ±10 μg m^{-3} at 25 °C and 50%RH	±10%@100~500 μg m^{-3} ±10 μ g m^{-3}@0~100 μg m^{-3}
Working temperature	-10 °C - +50°C	-10 °C - +60°C
Working relative humidity	0-70%	0-99%
Minimum resolution of particles	0.3 μm	0.3 μm
Size	71x70x23mm	38×35×12 mm
